# The Impacts of Short-Term NMN Supplementation on Serum Metabolism, Fecal Microbiota, and Telomere Length in Pre-Aging Phase

**DOI:** 10.3389/fnut.2021.756243

**Published:** 2021-11-29

**Authors:** Kai-Min Niu, Tongtong Bao, Lumin Gao, Meng Ru, Yumeng Li, Liang Jiang, Changming Ye, Shujin Wang, Xin Wu

**Affiliations:** ^1^Tianjin Institute of Industrial Biotechnology, Chinese Academy of Sciences (CAS), Tianjin, China; ^2^CAS Key Laboratory of Agro-Ecological Processes in Subtropical Region, Institute of Subtropical Agriculture, Chinese Academy of Sciences (CAS), Changsha, China; ^3^Institute of Biological Resources, Jiangxi Academy of Sciences, Nanchang, China; ^4^ERA Biotechnology (Shenzhen) Co., Ltd., Shenzhen, China; ^5^Institute of Life Sciences, Chongqing Medical University (CAS), Chongqing, China

**Keywords:** NMN, telomere, pre-aging, microbiota, metabolomic

## Abstract

Aging is a natural process with concomitant changes in the gut microbiota and associate metabolomes. Beta-nicotinamide mononucleotide, an important NAD^+^ intermediate, has drawn increasing attention to retard the aging process. We probed the changes in the fecal microbiota and metabolomes of pre-aging male mice (C57BL/6, age: 16 months) following the oral short-term administration of nicotinamide mononucleotide (NMN). Considering the telomere length as a molecular gauge for aging, we measured this in the peripheral blood mononuclear cells (PBMC) of pre-aging mice and human volunteers (age: 45–60 years old). Notably, the NMN administration did not influence the body weight and feed intake significantly during the 40 days in pre-aging mice. Metabolomics suggested 266 upregulated and 58 downregulated serum metabolites. We identified 34 potential biomarkers linked with the nicotinamide, purine, and proline metabolism pathways. Nicotinamide mononucleotide significantly reduced the fecal bacterial diversity (*p* < 0.05) with the increased abundance of *Helicobacter, Mucispirillum*, and *Faecalibacterium*, and lowered *Akkermansia* abundance associated with nicotinamide metabolism. We propose that this reshaped microbiota considerably lowered the predicated functions of aging with improved immune and cofactors/vitamin metabolism. Most notably, the telomere length of PBMC was significantly elongated in the NMN-administered mice and humans. Taken together, these findings suggest that oral NMN supplementation in the pre-aging stage might be an effective strategy to retard aging. We recommend further studies to unravel the underlying molecular mechanisms and comprehensive clinical trials to validate the effects of NMN on aging.

## Introduction

The aging population is continuously increasing. It has been predicted that the people over 60 years of age will be more than 1.2 billion globally in 2050 (1). Elongated lifespan, however, may come with undesirable health conditions, such as organ damage, metabolic dysfunction, decreased bone density, and untoward inflammatory responses (2). Indeed, aging can be slowed to a moderate decline of age-related functionality *via* intervening in the biological systems (viz., nutrient sensing, gut microbiome-targeted modulation) (3). Lifestyle adjustments like caloric restriction, time-restricted feeding, and alternate fasting, are known to improve cerebrovascular health in the elder population (4). Studies have shown that nicotinamide adenine dinucleotide (NAD^+^) levels decrease with aging in worms as well as mammals (5, 6). The inhibition of NAD^+^ consumption and/or replenishment of NAD^+^ precursors are considered to retard aging and age-predisposed diseases by boosting the NAD^+^ levels (7, 8). Nicotinamide mononucleotide is a key NAD^+^ precursor that has been deemed a potentially effective, affordable, and safe anti-aging agent capable of extending the lifespan and ameliorating age-related complications (8–10). Besides anti-aging activities, nicotinamide mononucleotide (NMN) also shows a variety of health benefits. Nicotinamide mononucleotide has displayed positive roles in angiogenic processes and anti-oxidative activities *via* the SIRT1-dependent signaling pathways (11, 12). The reduction of metabolic impairment in obese mice has been reported in NMN supplementation (13, 14). Enhanced intestinal homeostasis has been currently reported in NMN treatment *via* regulating the gut microbiota (15). An isotope labeling study has demonstrated that the gut bacteria compete with the host to consume orally delivered NMN (16).

Correlations have been observed between gut microbiota and age (17). Age-related microbial dysbiosis can induce intestinal permeability and systemic inflammation, further impacting late-life health (18). In addition, inflammations can perturb the balance of the gut microbiota, which in turn shortens the lifespan (19). Gut microbiota-targeted interventions have been conducted to retard aging and improve host health (20). A preventive effect of natural functional food on aging *via* the regulation of gut microbiota and relevant metabolites has also been demonstrated (21). Gut microbiota transplantation of young donors is evident to reverse age-associated impairments in the peripheral and brain immunity, and cognitive behavior in older recipients (22). Not only the gut microbiota has vital effects on aging, but also the microbial metabolites exhibit important roles in the lifespan. In recent years, metabolomics has been used to identify important biomarkers of healthy aging and longevity (23, 24). Additionally, an integrated metabolome-microbiome method displayed advances in the analysis of the relationship between host metabolism and gut microbiota (25). Huang and his colleagues have revealed that long-term oral NMN administration in drinking water increased the abundance of beneficial microbes and contents of bile acid-related metabolites by combining fecal microbiome and metabolomic analysis (15).

Telomere length is an important aging biomarker that reduces during aging (26). The administration of NMN has been proved to maintain telomere length in the liver of mice (27). In aging studies, murine (*Mus musculus*) is one of the most widely used experimental models. Jackson laboratory has defined the age stages of C57BL/6J mice as mature adults (3–6 months), middle-aged (10–14 months), and old (18–24 months) which have been corresponded to the human age of 20–30 years old (mature adult), 38–47 years old (middle-aged), and 56–69 years old (old), respectively (28). Research in aging is generally performed with mice not <18 months and human volunteers not <60 years old (29). Herein, we designated a pre-aging stage between the middle-aged and mature adults to about 15–17 months for mice and 45–60 for human volunteers. We investigated whether supplementing the NMN at a pre-aging stage could slow down the aging process. Herein, we attempted to investigate the effects of short-term NMN supplementation on the serum metabolites and gut microbiota in the pre-aging mice, as well as the telomere length in both the pre-aging mice and humans enrolled in the present study.

## Materials and Methods

### Animals and Experimental Design

The animal experiment was approved and conducted following the Regulations and Administration of the Committee of the Institute of Subtropical Agriculture at the Chinese Academy of Science (No.ISA-2020-18). A total of twenty 16-month-old male C57BL/6 specific pathogen-free mice (STA Laboratory Animal Co., LTD, Hunan, China) were used in the study. All the mice were housed with two mice per cage and raised under controlled conditions (temperature 25 ± 2°C, light/dark 12 h:12 h, humidity 60 ± 10%). The mice had free access to feeds and water. After 4 days of acclimatization, the mice were randomly assigned as the control group and NMN supplemented group with five replicates in each treatment (a cage/replicate). All the mice were fed a chow diet (D12450J, 17.70 kJ/g), which was purchased from the SLAC Laboratory Animal Central (Changsha, China). The control group mice were fed with water, and the NMN group mice were fed with water containing 500 mg/L (w/v) of NMN. The NMN dosage accepted in the experiment was referred to a previous study (15). The NMN-containing water bottles and cages were changed weekly. The whole experiment lasted for 40 days, the food intake and body weight were measured every 5 days, and the water intake was measured every 7 days.

### Heat Yield Measurement

The heat yield of the mice was measured by an infrared camera (Seek Thermal Compact XR iOS Camera, Seek Thermal, Inc., CA, USA) at end of the experiment. The pixels of the images were measured using Image J v1.8.0, National Institutes of Health, Bethesda, Maryland, USA and presented by a histogram.

### Sample Preparation

At end of the experiment, all the mice were fasted for 6 h. The fresh feces of all the mice were directly collected from the anus of the mice to analyze the fecal microbiota. The mice were induced with anesthesia by the intraperitoneal injection of 2% pentobarbital sodium (45 mg/kg body weight). The blood was taken from the enucleation of the eyeballs and collected into a 1.5 ml sterile Eppendorf tube (Eppdendorf, Hamburg, Germany), and then placed at room temperature for 30 min. The blood samples were centrifuged for 15 min at 3,000 *g* and 4°C, the serum was collected and stored at −80°C until further analyses.

### Serum Metabolomics

The serum metabolites were determined by a commercial service in the Biotree company (Shanghai, China). Briefly, 50 μl of the serum sample was mixed with 200 μl of extracting solution containing 50% methanol and 50% acetonitrile and the two internal standards (L-leucine-5,5,5-d3, CAS:87828-86-2, trimethylamine-d9-N-oxidein, CAS: 1161070-49-0), followed by 10 min of sonication under iced conditions, and then was centrifuged for 15 min at 11,000 *g* and 4°C to collect the supernatant. The supernatant was subjected to Vanquish ultra-high performance liquid chromatography-mass spectrometry (UHPLC-MS) platform (Thermo Fisher Scientific, Massachusetts, United States) with an ACQUITY UPLC BEH Amide (2.1 × 100 mm, 1.7 μm) chromatographic column and Q Exactive HFX mass spectrometer (Orbitrap MS, Thermos) and scanned for the positive model. The injection volume was 3 μl. The mobile phase used in the liquid chromatography (LC) elution includes solvent A (25 mmol/L ammonium acetate and 25 mmol/L ammonia in ultrapure water) and solvent B (acetonitrile) with the elution gradient as follows: 0–.5 min, 95% B;.5–7 min, 95–65% B; 7–8 min, 65–40% B; 8–9 min, 40% B; 9–9.1 min, 40–95% B; 9.1–12.0 min, 95% B. The full scan mass spectrum was obtained based on the information-dependent acquisition (IDA) mode in the control of the acquisition software (Xcalibur, Thermo Fisher Scientific). The electron spray ionization source conditions with 50 Arb sheath gas flow rate, 10 Arb Aux gas flow rate, 320°C capillary temperature, 60,000 full mass spectrometry (MS) resolution, 7,500 MS/MS resolution, 10/30/60 collision energy in normalized collisional energy mode, and 3.5 kV spray Voltage (positive) were used. For the data analysis, ProteoWizard database, Palo Alto, CA, USA was used to convert the raw data to the mzXML format, and then processed with an in-house R software, and (X) of chromatography mass spectrometry was used to detect, extract, align and integrate the peak. The peaks were normalized using an internal standard. The principal component analysis (PCA) and orthogonal partial least squares discriminant analysis (OPLS-DA) were performed using SIMCA-P (16.0.2, Sartorius Stedim Data Analytics AB, Umea, Sweden) to cluster the sample plots across groups. To screen the significantly different metabolic markers, univariate statistical analysis was used based on the criteria of variable importance in the projection (VIP) >1 and the fold change of metabolites <0.5 or more than 2, coupling with *p*-value <0.05, which was visualized by a volcano plot and heatmap plot. The relevant significant changed metabolism pathway was determined based on the database of Kyoto Encyclopedia of Genes and Genomes (KEGG).

### Fecal DNA Extraction and Sequencing

Fecal samples were freshly collected and snap-frozen using liquid nitrogen and stored at −80°C. The bacterial genomic DNA was extracted using the CTAB method. The concentration was measured using a NanoDrop 2000, the purity and quality of the genomic DNA were checked by running 1% agarose gel electrophoresis. The V4 hypervariable regions of the 16S ribosomal RNA (rRNA) were amplified using 515F and 806R primers. The PCR conditions were 98°C for 1 min, followed by 30 cycles of denaturation at 98°C for 10 s, annealing at 50°C for 30 s, and elongation at 72°C for 30 s. The PCR products were purified using a Qiagen gel extraction kit (Qiagen, Germany). The amplicons were sequenced using an Illumina NovaSeq 6000 platform by a commercial service of Novogene Bioinformatics Technology Co., Ltd (Beijing, China).

### Fecal Microbial Analyses

For the sequence analysis, UParse software (Uparse v7.0.1001, http://drive5.com/uparse/) was used. The sequences of similarity ≥97% were assigned to the same operational taxonomic units (OTUs) (30). The Silva database (http://www.arb-silva.de/) with the Mothur algorithm was used to annotate the taxonomic information of the representative sequences (31). The alpha diversity including observed_species, Chao1, Simpson, and Shannon was determined in QIIME (version 1.7.0) open source software (http://qiime.org/) and visualized by R software (version 2.15.3). The beta diversity includes PCA based on the OTU level, principal coordinate analysis (PCoA) based on the unweighted unifrac matrix, and non-metric multi-dimensional scaling (NMDS) based on Bray–Curtis distance were calculated in QIIME (Version 1.9.1.). Tax4Fun R package (http://tax4fun.gobics.de/) was used to analyze the predicated functions of bacterial species (32).

### DNA Isolation and Measurement of Telomere Length

A non-blinded clinical trial in eight healthy men was conducted to investigate the supplementary effect of NMN on the telomere length of the peripheral blood mononuclear cell (PBMC). The male subjects enrolled in the study were selected based on the criteria based on an NMN clinical trial NCT04228640 (https://clinicaltrials.gov/ct2/show/study/NCT04228640) and a previous study (33) as follows: (1) 45–60 years old with body mass index (BMI) at a range of 18.5–30 kg/m^2^; (2) no allergic and metabolic diseases; (3) without any form of niacin supplement for 7 days prior to the study and for the whole test period; (4) kept consistent diet and lifestyle habits during the whole test period; (5) took NMN supplement for 90 days; (6) followed verbal and written study directions. The information of the volunteers is presented in Supplementary Table 1. All the participants were instructed to take NMN (300 mg/day/person) (34) in warm water once a day after 30 min of breakfast for a total of 90 days. The blood of all the participants was taken by a doctor at 0, 30, 60, and 90 days of NMN administration using ethylene diamine tetraacetic acid-containing anticoagulant tubes. The blood was separated into serum and PBMC for the analyses of serum cholesterol, triglyceride, and glucose contents using an automatic biochemical analyzer (Beckman Coulter AU5811) with commercial kits. The genomic DNA was extracted from the PBMC from the whole blood of mice and human samples using a FastPure Blood DNA Isolation Mini Kit V2 (Vazyme, Nanjing Vazyme Biotech Co., Ltd, China) following the manual of the manufacturer. The DNA concentration was determined using a NanoDrop 2000 spectrophotometer ThermoFisher Scientific, Waltham, MA, USA. Real-time quantitative PCR was used to assess the telomere length (TL) following the previously described methods (35, 36). Briefly, the primers of tel1b, F-CGGTTTGTTTGGGTTTGGGTTTGGGTTTGGGTTTGGGTT, and te12b R-GGCTTGCCTTACCCTTACCCTTACCCTTACCCTTACCCT was used to amplify the telomeres (T) of the mouse and human. 36B4 primers (mouse: F-ACTGGTCTAGGACCCGAGAAG and R-TCAATGGTGCCTCTGGAGATT; human: F-CAGCAAGTGGGAAGGTGTAATCC, R-CCCATTCTATCATCAACGG-GTACAA) were used to amplify the single-copy gene (S). The relative TL was measured by comparing the ratio of T repeat copy number and S copy number, expressed as the telomere length (T/S) ratio. The clinical study was reviewed and approved by the Institute of Life Sciences, Chongqing Medical University, Chongqing. Verbal and written informed consents were obtained from each subject before the clinical study.

### Statistics

Statistical analysis was conducted using Prism GraphPad 7 software (GraphPad Software Inc., San Diego, California, United States). The significant difference between the control group and the NMN group were performed based on the student's *t*-test with non-parametric tests.

## Results

### NMN Supplementation Enhanced Heat Yield in Pre-Aging Mice

**Figure 1:**
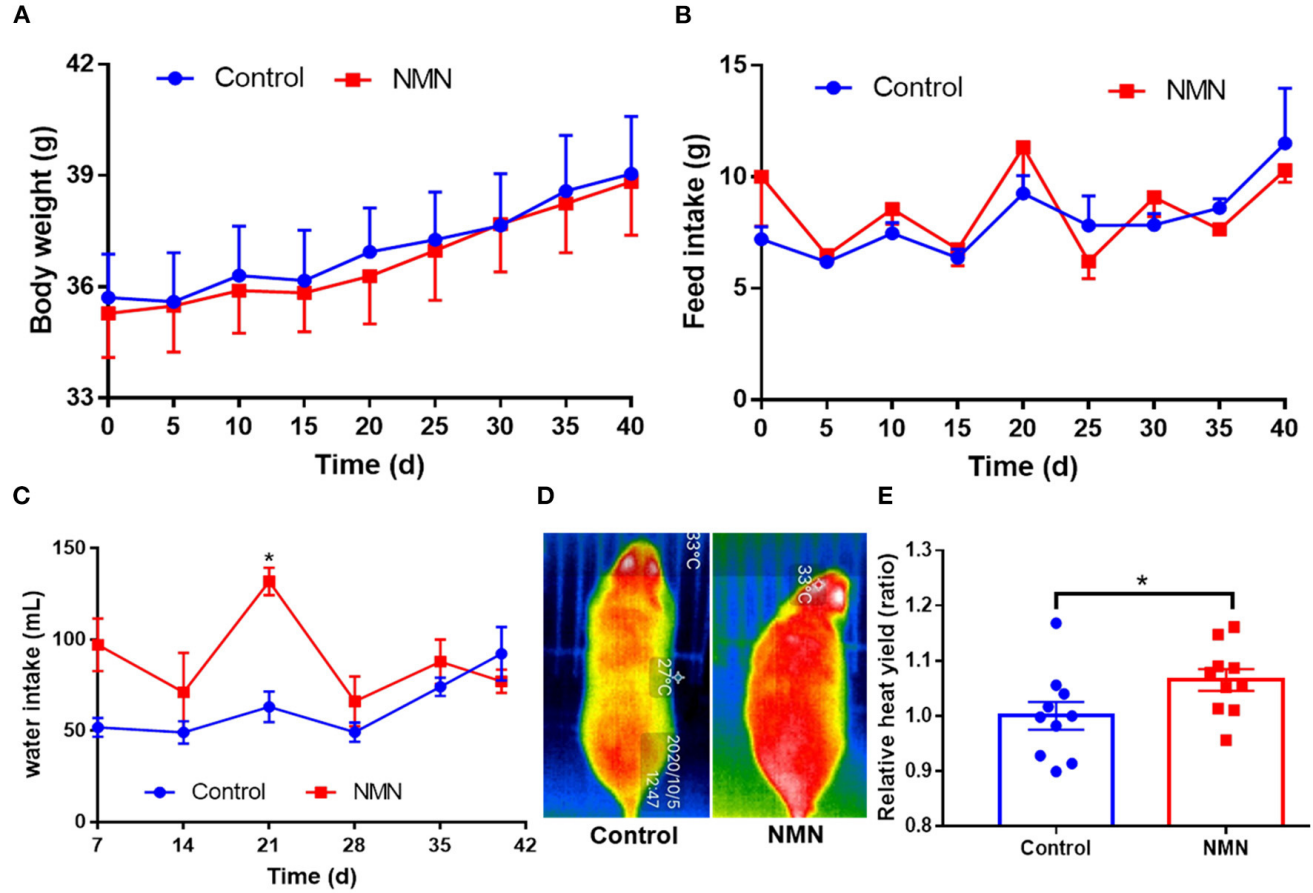
Supplementary effects of nicotinamide mononucleotide (NMN) on the body weight **(A)**, feed intake **(B)**, water intake **(C)**, heat yield image **(D)**, and histogram **(E)** of pre-aging mice. Values indicate mean ± SEM (*n* = 10). *Indicates significant difference at *p* < 0.05 level.

The supplementary effect of NMN on age-associated body weight change was determined. In comparison with the control mice, the NMN-supplemented mice showed little change in body weight and feed intake (Figures 1A,B), but significantly increased the water intake (Figure 1C) on 21 days (*p* < 0.05). Moreover, the NMN-supplemented mice significantly increased the heat yield after 40 days (*p* < 0.05) (Figures 1D,E).

### Effect of NMN Supplementation on Serum Metabolome in Pre-Aging Mice

**Figure 2:**
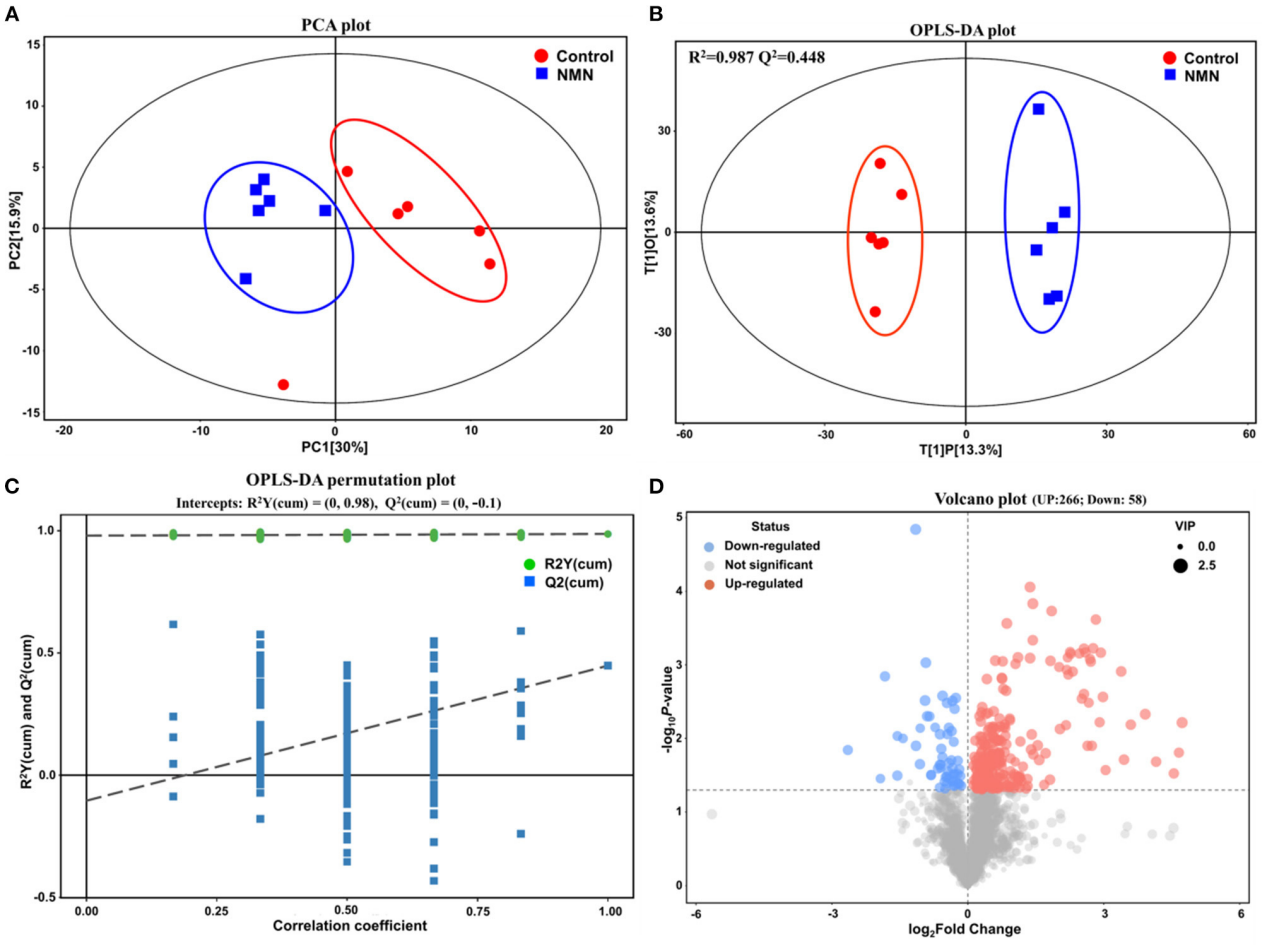
Supplementary effect of NMN on serum metabolome in pre-aging mice by multivariate statistical analysis. **(A)** principal component analysis (PCA) plot, **(B)** orthogonal partial least squares discriminant analysis (OPLS-DA) score plot, **(C)** OPLS-DA permutation test plot, and **(D)** Volcano plot.

**Figure 3:**
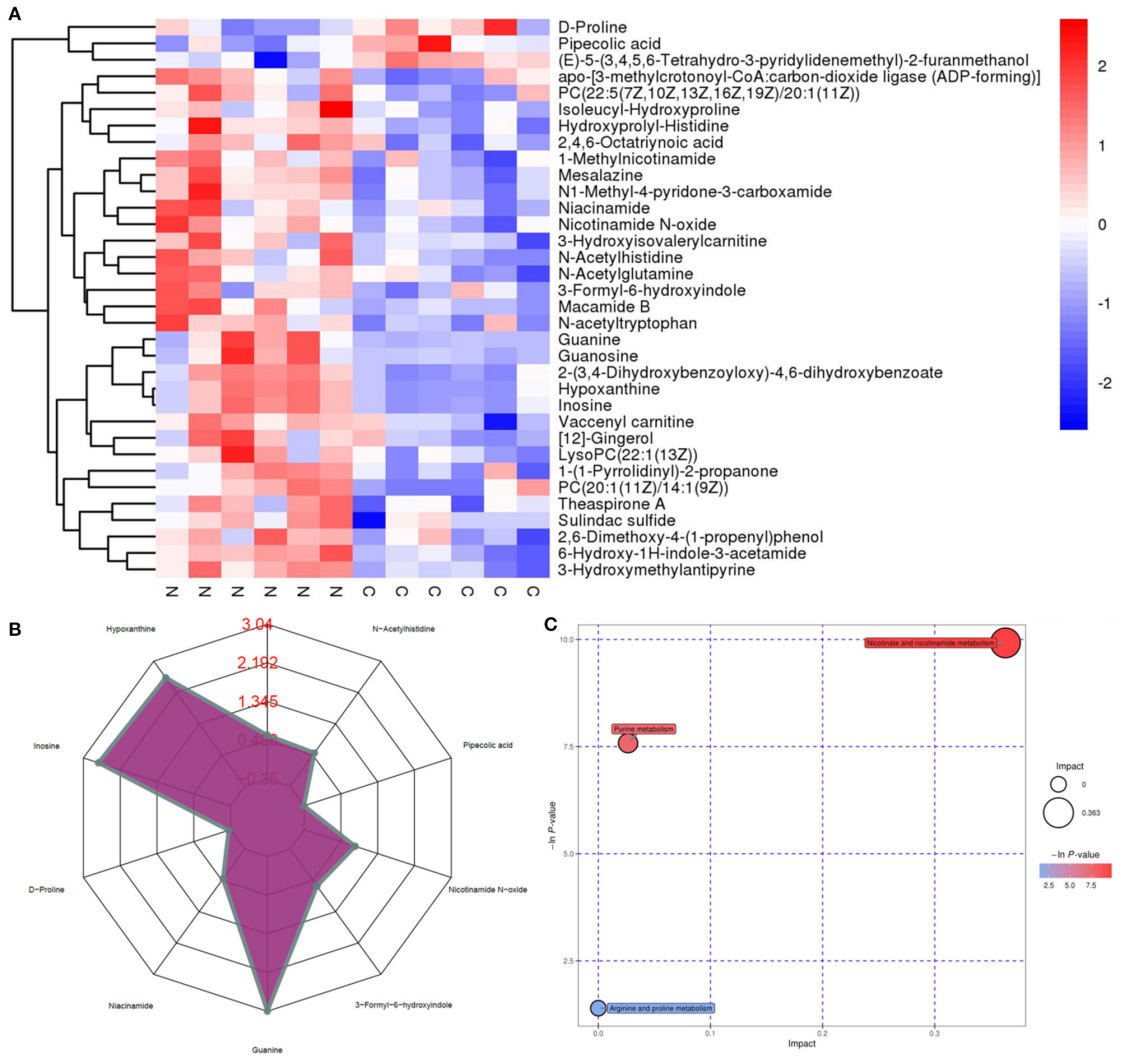
Supplementary effect of NMN on the top 34 metabolic biomarkers and main metabolic pathways in pre-aging mice by hierarchical clustering analysis. **(A)** heatmap of hierarchical clustering analysis, **(B)** radar chart, and **(C)** pathway analysis (NMN group vs. control group).

Untargeted metabolomics was performed to analyze the effects of NMN supplementation on serum metabolites in pre-aging mice. The PCA and OPLS-DA results showed that the metabolite profiling datasets were clustered separately between the NMN group and the control group (Figures 2A,B). The permutation test indicated that the OPLS-DA model was reliable without overfitting (Figure 2C). The volcano plot displayed significant changes in the serum metabolite profiles for the NMN group. Overall, 266 metabolites were upregulated and 58 downregulated as compared with those in the control group (Figure 2D). Thirty-four significantly discriminant biomarker metabolites were selected based on the VIP value >1 and *p*-value <0.05 using the OPLS-DA model (Figure 3A). Specifically, among these metabolites, D-proline, pipecolic acid, and (E)-5-(3,4,5,6-Tetrahydro-3-pyridylidenemethyl)-2-furanmethanol were down-regulated, while hypoxanthine, inosine, guanine, 1-Methylnicotinamide, N1-Methyl-4-pyridone-3-carboxamide, niacinamide, nicotinamide N-oxide, 3-Formyl-6-hydroxyindole, N-acetylhistidine, N-acetyltryptophan, and 6-Hydroxy-1H-indole-3-acetamide were upregulated in the NMN group compared to the control group (Figures 3A,B). Notably, these discriminant metabolites were associated with metabolic pathways encompassing nicotinate/nicotinamide metabolism, purine metabolism, and arginine/purine metabolism (Figure 3C).

### Effect of NMN Supplementation on Fecal Microbiota in Pre-Aging Mice

**Figure 4:**
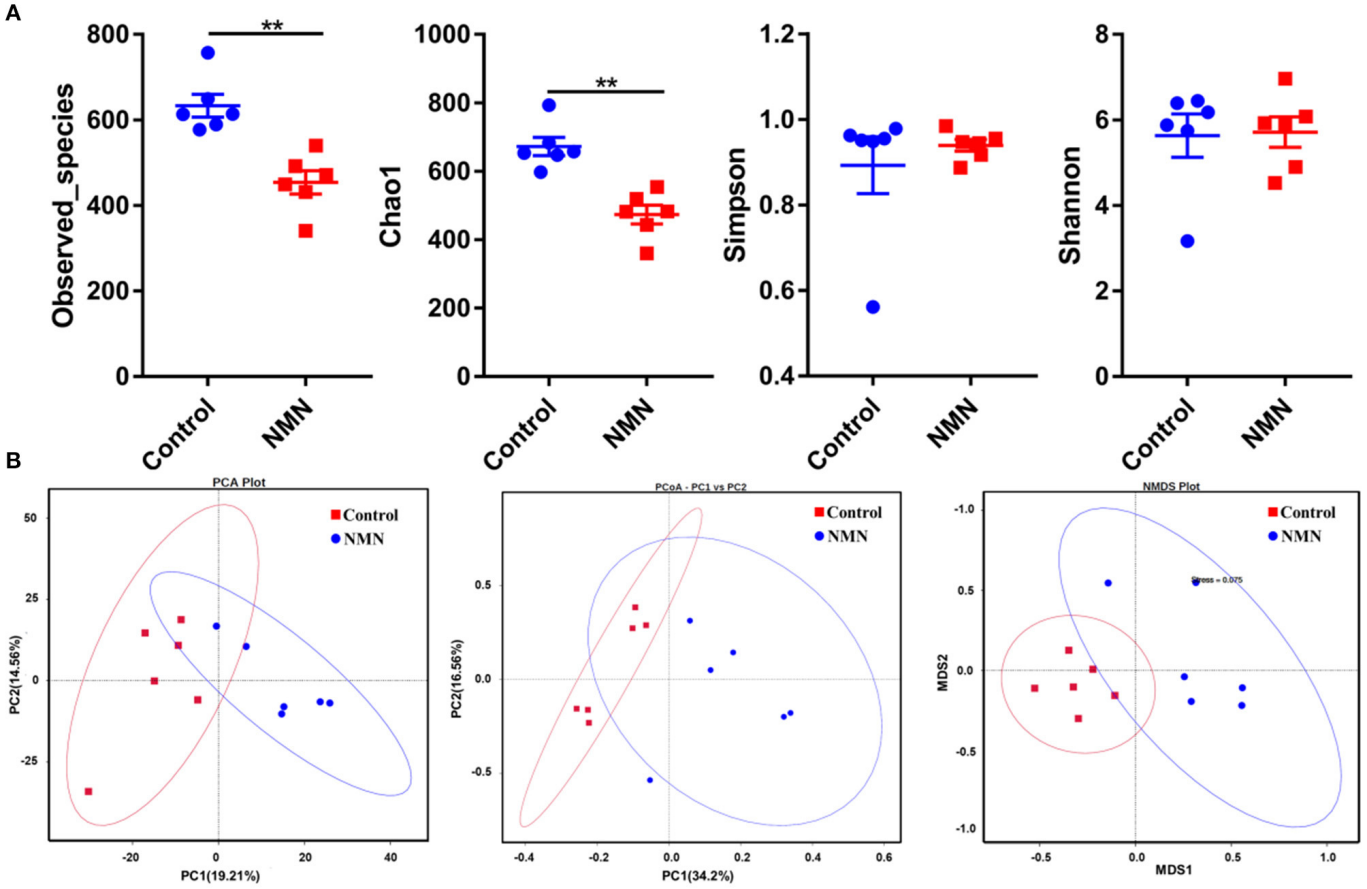
Supplementary effect of NMN on the fecal microbiota in pre-aging mice. **(A)** Alpha diversity indexes, **(B)** Beta diversity indexes. **Indicates significant difference at *p* < 0.01.

**Figure 5:**
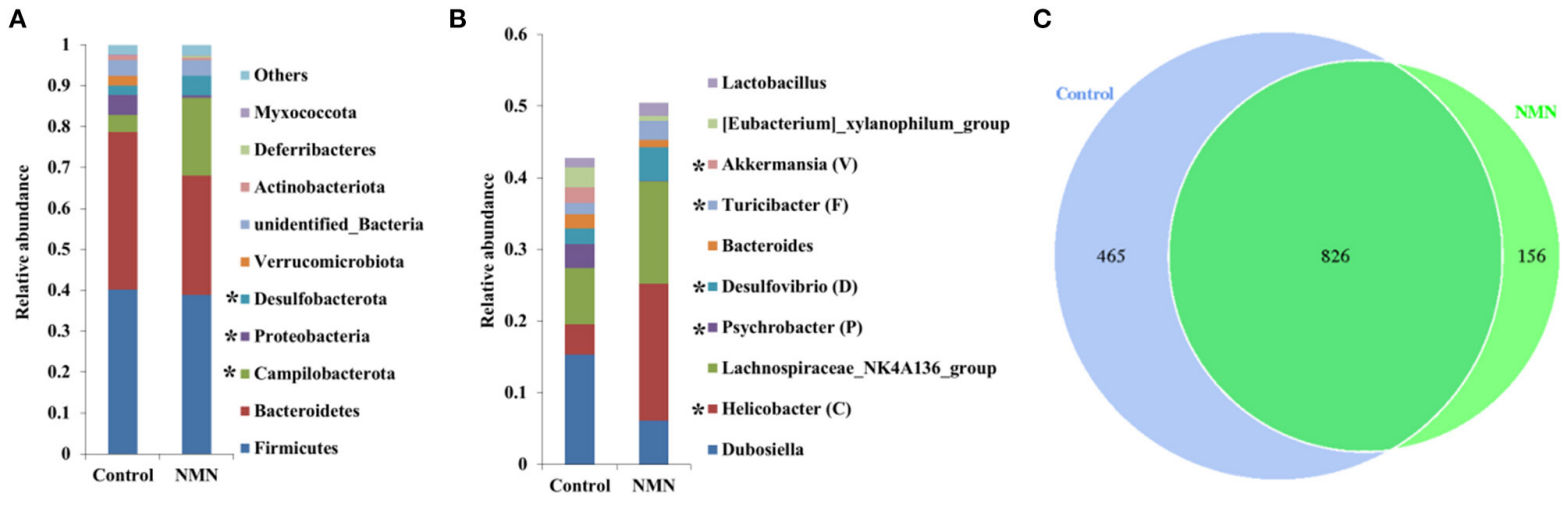
Supplementary effect of NMN on dominant fecal bacteria in pre-aging mice. **(A)** Phylum level, **(B)** genus level, and **(C)** venn diagram. *Indicates significant difference at *p* < 0.05 level.

**Figure 6:**
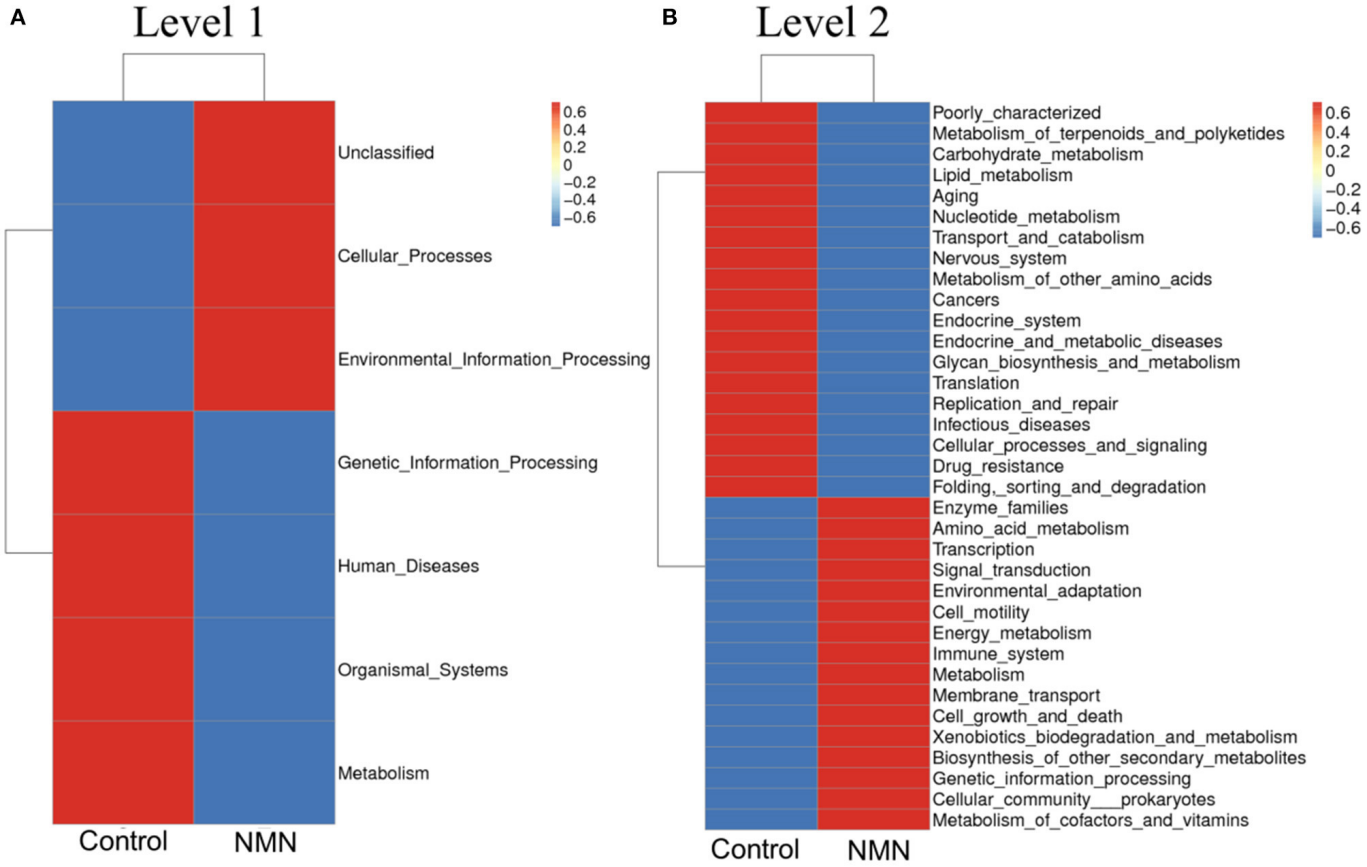
Supplementary effect of NMN on predicted functions of fecal bacteria in pre-aging mice. **(A)** level 1, **(B)** level 2.

The 16S ribosomal DNA (rDNA) gene sequencing was employed to investigate the effect of NMN supplementation on the fecal microbiota diversity and composition in pre-aging mice. The NMN supplementation significantly lowered the alpha diversity of fecal microbes based on the observed species and Chao1 α-diversity indexes (Figure 4A). The β-diversity was analyzed by performing PCA, PCoA, and NMDS plots based on the unweighted unifrac matrix, which displayed distinctly separated fecal microbiota (Figure 4B). The dominant phyla were Firmicutes, Bacteroidetes, Camilobacterota, Proteobacteria, and Desulfobacterota (Figure 5A), and the dominant genera were *Dubosiella, Helicobacter, Lachnospiraccae*_NK4A136_group, and *Psychrobacter* (Figure 5B). Of these, NMN supplementation significantly enriched the abundance of Camilobacterota and Desulfobacterota phyla, and *Helicobacter, Desulfovibrio*, and *Turicibacter* genera, and reduced the abundance of Proteobacteria phylum and *Psychrobacter* and *Akkermansia* genera. In addition to these dominant genera, NMN supplementation also enriched *Mucispirillum, Colidextribacter, Candidatus_Saccharimonas, Marvinbryantia, Faecalibacterium*, unidentified_*Oscillospiraceae*, A2, UCG-009, *Oscillibacter*, and *Lachnospiraceae*_UCG-001, but reduced the abundance of *Staphylococcus, Corynebacterium*, and *Paenalcaligenes* (Supplementary Table 2). Notably, a total of 826 core existent species were identified, while 465 and 156 unique species were observed in the control group and NMN group, respectively (Figure 5C). The predicted functional analysis further showed that NMN supplementation significantly downregulated the metabolism and human disease-related functions (at level 1), which are mainly associated with carbohydrate metabolism, lipid metabolism, glycan biosynthesis/metabolism, aging, cancers, and infectious disease (at level 2). On the other hand, NMN supplementation significantly enhanced the cellular processes and environmental information processing functions (at level 1), which are mainly involved in amino acid metabolism, energy metabolism, cofactors/vitamins metabolisms, environmental adaptation, immune system, and xenobiotics biodegradation/metabolism (at level 2) (Figure 6).

### Correlation Analysis of Serum Metabolome and Fecal Microbiota With NMN Supplementation in Pre-Aging Mice

**Figure 7:**
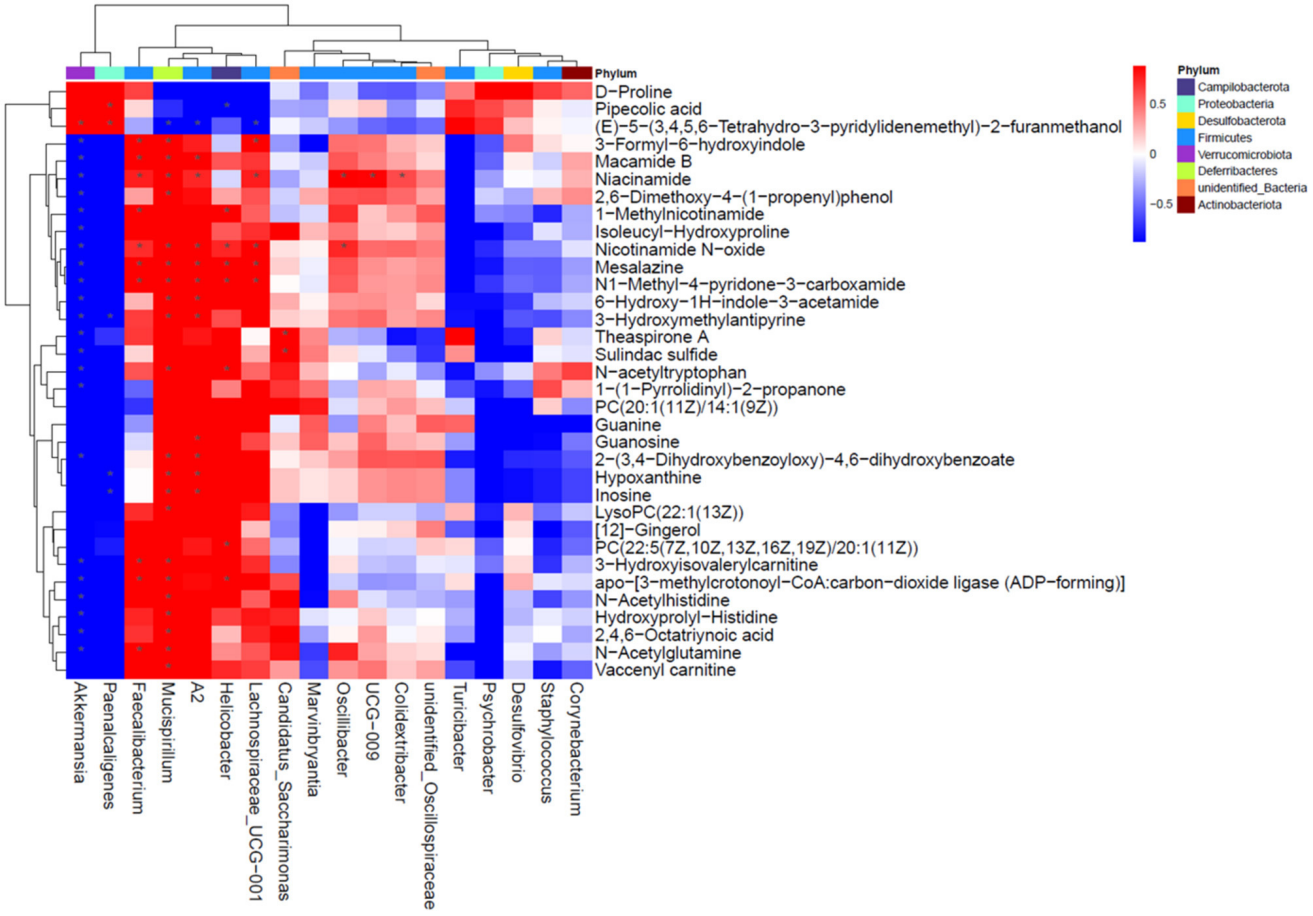
Correlation analysis for differentially changed bacteria and metabolites with NMN supplementation in pre-aging mice.

Based on the results of the serum metabolite and 16S rDNA sequencing results, the Spearman correlation analysis was performed to explore the association between the top 18 significantly changed bacteria (Supplementary Table 2) and 34 differentially changed serum metabolites (Figure 7). Among these bacteria, *Akkermansia, Faecalibacterium, Mucispirillum*, A2, *Helicobacter*, and *Lachnospiraceae* were closely correlated to the varied metabolites. The *Akkermansia* genus was positively correlated with pipecolic acid and (E)-5-(3,4,5,6-Tetrahydro-3-pyridylidenemethyl)-2-furanmethanol, and negatively correlated with the other metabolites. *Faecalibacterium, Mucispirillum*, A2, *Helicobacter*, and *Lachnospiraceae* were positively correlated with niacinamide, nicotinamide N-oxide, mesalazine, and N1-methyl-4-pyridone-3-carboxamide, those of which are linked with nicotinate and nicotinamide metabolism. Moreover, *Mucispirillum* and A2 were also significantly positively correlated with hypoxanthine and inosine that are involved in purine metabolism. Taken together, the result showed that the changed bacterial structure could impact the composition of serum metabolite constitutes.

### NMN Supplementation Elongates Telomere Length in Pre-Aging Mice and Humans

**Figure 8:**
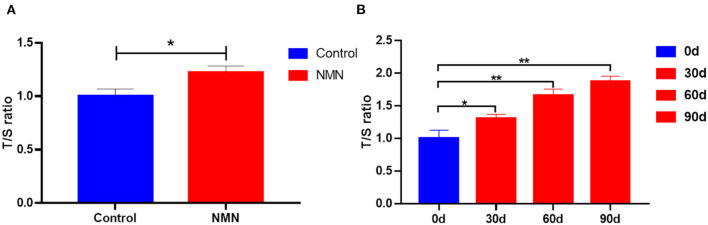
Supplementary effect of NMN on the telomere length of the peripheral blood mononuclear cell (PBMC) in **(A)** pre-aging mice (*n* = 6), and **(B)** pre-aging human volunteers (*n* = 8). *Indicates significant difference at *p* < 0.05. **Indicates significant difference at *p* < 0.01.

The TL of PBMC was measured using a PCR-based method. In the pre-aging mice, the length of telomere was significantly increased with 40 days of NMN supplementation (Figure 8A). A similar result was also observed in pre-aging human volunteers after 30 days of NMN supplementation (Figure 8B).

## Discussion

In the present study, NMN administration did not affect the body weight and feed intake of the pre-aging mice during the experimental period. Similar results have been observed in young mice supplemented with 0.1–0.6 mg/ml NMN in their drinking water (15). However, Mills et al. have reported that the administration of 300 mg/kg/day NMN in drinking water mitigated age-associated body weight gain, enhanced food and water intake, and energy expenditure (9). The higher heat yield in NMN-supplemented mice in the present study also indicates that the NMN administration possibly accelerated thermogenesis.

Aging is accompanied by changes in amino acid, lipid, sugar, hormone, and nucleotide metabolism (37). Eighteen months is considered as the lower limit of “Old” or “Aging” mice as almost all aging-related biomarkers will be detected by then (28). To the best of our knowledge, no studies have reported the impact of short-term NMN supplementation on the serum metabolites and gut bacterial community in the pre-aging phase (15–17 months) of mice. In the present study, NMN supplementation notably altered the serum metabolites that mainly clustered with the metabolism pathways in terms of purine, nicotinate/nicotinamide, and arginine/proline in pre-aging mice. Previously, Houtkooper et al. have reported that the levels of long-chain acylcarnitines and amino acids decrease considerably in aged mice (38).

In a previous study, increased proline levels coupled with decreased inosine and histamine contents have also been reported for aged *Drosophila* as compared with young flies (39). In the present study, the decreased D-proline coupling with the increased inosine, hydroxyprolyl-histidine, and acetyl histidine metabolites in NMN-supplemented pre-aging mice implies that NMN might have restored some lost metabolites in the aged hosts. Collino et al. indicated that the level of tryptophan is decreased following the aging process (40). In our study, NMN administration enriched the serum indole derivatives (i.e., 3-Formyl-6-hydroxyindole and 6-Hydroxy-1H-indole-3-acetamide) and N-acetyltryptophan. Indoles from commensal microbiota are evident to extend the healthy lifespan of *Caenorhabditis elegans, Drosophila melanogaster*, and mice (41). The tryptophan-derived synthesis of NAD^+^ is identified as a *de novo* pathway (34). Herein, the effect of exogenous NMN on the synthesis of NAD^+^ *via* the acetylation of tryptophan remains unclear. Furthermore, we also found that the nicotinamide metabolism pathway-related 1-Methylnicotinamide, N1-Methyl-4-pyridone-3-carboxamide, niacinamide, and nicotinamide N-oxide were enriched in the serum of the mice who ingested NMN. It is known that NMN replenishment can increase the cellular NAD^+^ level, then NAD^+^ can be converted into niacinamide (nicotinamide, Nam) which is further metabolized to nicotinamide N-oxide by cytochrome P450 or transformed to 1-Methylnicotinamide (MNA) by nicotinamide N-methyltransferase. Then, MNA can be further metabolized to 1-methyl-2-pyridone-5-carboxamide (Me2PY) or 1-methyl-4-pyridone-5-carboxamide (Me4PY) by aldehyde oxidase (10, 33). In one clinical study, NMN administration significantly increased the serum MNA and Me4PY in healthy men (33).

The complex interplay between age and the microbiota is well-described in several studies (17, 18, 20). The changes in the composition, diversity, and functional characters of the microbiota were observed over time. Luo et al. reported that the abundance of Proteobacteria is positively linked with aging (25). Proteobacteria include pathogenic representatives, such as *Enterobacter* spp., which may cause infection and disease (25, 42). In the present study, the reduced abundance of fecal Proteobacteria in the NMN-supplemented mice suggests that NMN might have perturbed certain harmful microbes. Surprisingly, a widely accepted probiotic strain *Akkermansia* (Verrucomicrobiota phylum) was lowered in the fecal microbiota of NMN-supplemented mice. In contrast, a previous study has reported that NMN administration enriches the abundance of *Akkermansia muciniphila* (15). We conjecture that the observed differences in the outcomes may be attributed to the difference in the age of the mice used in the experiments. We clearly observed that the *Akkermansia* abundance was negatively correlated to nicotinamide, tryptophan, and indole as well as their derivatives, which might have inhibited its growth. Nicotinamide mononucleotide increases the abundance of butyric acid-producing *Turicibacter* which exhibits anti-fatigue activity (43), implying that NMN administration might reinforce vitality by promoting the growth of *Turicibacter*. Unexpectedly, in this study, oral NMN administration increased *Helicobacter* abundance in pre-aging mice. Some *Helicobacter* spp. are known as pathogenic bacteria that can cause gastric diseases (44), its enrichment with NMN administration should be deeply and carefully confirmed further. In addition to these top dominant genera, the correlation analysis demonstrated that *Mucispirillum* was greatly associated with the altered serum metabolites. *Mucispirillum* was positively correlated with the metabolites relevant to purine, nicotinate, and nicotinamide metabolism, as well as arginine and proline metabolism. *Mucispirillum schaedleri* showed a protective effect against *Salmonella enterica* ser. Typhimurium colitis by interfering with the invasion gene expression (45). These upregulated metabolites might have beneficial effects on the inhibition of pathogenic adhesion in the gut mucus. It is not yet fully understood how these metabolites change with the varied microbial composition in response to the NMN supplementation. Further validation of specific metabolite changes corresponding to specific microbial genera coupled with their downstream biological effects will be important to the effects of NMN supplementation on the host.

Telomeres are nucleoprotein complexes composed of several kilobases of TTAGGG repeats and located at the end of eukaryotic chromosomes that protect the stability of chromosomes from recombination (46). Telomere length has been documented as an important feature of aging (26). Ghimire et al. have studied the correlation between TL and age from the data of 7,826 adults based on the National Health and Nutrition Examination Survey (years 1999–2002, age from >20 to >80) and found that a shorter telomere length is related to aging (47). In the present study, we observed longer T in pre-aging volunteers with NMN administration, which suggests the potential molecular mechanisms of NMN-mediated improved lifespan. In pre-aging mice, a similar effect has also been observed. In an animal model, Antonini et al. have also shown that the telomere length of isolated PBMCs was significantly decreased with aging in different male rat strains (Sprague-Dawley, F344, and Brown Norway) (48). Notably, NMN administration has been found to maintain TL and dampen the DNA damage response in the livers of telomerase knockout mice (27). In addition, the mice derived from embryonic stem cells with hyper-long T showed less DNA damage and weight, as well as improved glucose and insulin tolerance (49). The underlying mechanism of NMN on increasing telomere length might be associated with the increased NAD^+^ level (50), which in turn stabilizes T and prevent tissue damage and fibrosis in a partially sirtuin-1-dependent manner (27). However, whether altered microbiota and/or metabolomic are associated with elongated T is still unclear.

## Conclusions

We conclude that the short-term NMN supplementation did not significantly alter the bodyweight of the pre-aging mice. Serum biomarkers encompassing D-proline, inosine, 1-Methylnicotinamide, N1-Methyl-4-pyridone-3-carboxamide, niacinamide, nicotinamide N-oxide, 3-Formyl-6-hydroxyindole, N-acetylhistinde, N-acetyltryptophan, and 6-Hydroxy-1H-indole-3-acetamide were identified and mainly involved in the purine, nicotinate/nicotinamide, and arginine/proline metabolism pathways. Moreover, NMN supplementation notably altered the fecal microbial community. The varied functional bacteria associated with some specific serum metabolites could be potential biomarkers to estimate the anti-aging effects of NMN with further validation. Further, we observed longer T in both the mice and volunteers with NMN supplementation and reported for the first time that NMN could elongate the length of the T in a clinical study, suggesting the potential of NMN use at a pre-aging phase to retard the proceeding of aging.

However, there are certain limitations to the present study which pave the way to study broader realms of this concept. We used untargeted metabolomics to screen the altered metabolites related to aging at a wide range, however, targeted metabolomics is needed to quantify the changes in specific biomarkers. Further, owing to the finite amount of blood samples available, other important biochemical parameters could not be analyzed. For the clinical study, we did not perform a placebo-controlled trial and only enrolled a small number of subjects. Even the dosage-dependent NMN has been shortly administered to 10 healthy men in a clinical study and reported without causing any marked deleterious effects (33), but the safety and efficacy of the long-term NMN administration should be further investigated. The blood NMN metabolism and fecal microbiota were not measured in the clinical study. We are planning a more comprehensive study to explore the impact of NMN on gut health of aging mice, particularly focusing on *Helicobacter* and *Akkermansia* abundance, supported by cross-sectional clinical trials.

## Data Availability Statement

The datasets generated and analyzed for fecal microbiota in this study can be found in NCBI, BioProject accession number: PRJNA752982.

## Ethics Statement

The studies involving human participants were reviewed and approved by Regulations and Administration of the Committee of Chongqing Medical University. The patients/participants provided their written informed consent to participate in this study. The animal study was reviewed and approved by Regulations and Administration of the Committee of the Institute of Subtropical Agriculture at the Chinese Academy of Science (No. ISA-2020-18). Written informed consent was obtained from the individual(s) for the publication of any potentially identifiable images or data included in this article.

## Author Contributions

K-MN: formal analysis, writing the original draft, writing, review, and editing of the revised manuscript. TB, LG, MR, and YL: investigation, formal analysis, validation, review, and editing the manuscript. LJ and CY: conception of the idea, investigation, review, and editing the manuscript. SW: review and editing of the manuscript. XW: conception of the idea, investigation, review, editing of the revised manuscript, administration-lead, and supervision-lead. All authors contributed to the article and approved the submitted version.

## Funding

This paper was jointly supported by grants from the Tianjin Synthetic Biotechnology Innovation Capacity Improvement Project (SBICIP-CXRC-031), Science and Technology Projects of Hunan Province (2019RS3020), and Jiangxi Provincial Innovation and Entrepreneurship projects.

## Conflict of Interest

LJ and CY were employed by ERA Biotechnology (Shenzhen) Co., Ltd. The remaining authors declare that the research was conducted in the absence of any commercial or financial relationships that could be construed as a potential conflict of interest.

## Publisher's Note

All claims expressed in this article are solely those of the authors and do not necessarily represent those of their affiliated organizations, or those of the publisher, the editors and the reviewers. Any product that may be evaluated in this article, or claim that may be made by its manufacturer, is not guaranteed or endorsed by the publisher.
