# Supplementary material for: The Impacts of Short-Term NMN Supplementation on Serum Metabolism, Fecal Microbiota, and Telomere Length in Pre-Aging Phase
Source: Front Nutr. 2021 Nov 29;8:756243. doi: 10.3389/fnut.2021.756243 (PMC8667784; doi:10.3389/fnut.2021.756243)
Supplement: Supplementary file 1 [file Table_1.DOCX]

**Supplementary information**

**Table S1**. Information of healthy volunteers joined in the present study.

| Name | Gender | Age | Body weight (kg) | Height (cm) | BMI index |
| --- | --- | --- | --- | --- | --- |
| Participant 1 | Male | 55 | 70 | 167 | 25.10 |
| Participant 2 | Female | 55 | 72 | 167 | 25.82 |
| Participant 3 | Male | 51 | 72 | 167 | 25.82 |
| Participant 4 | Female | 55 | 58 | 159 | 22.94 |
| Participant 5 | Male | 48 | 70 | 169 | 24.51 |
| Participant 6 | Male | 54 | 73 | 172 | 24.68 |
| Participant 7 | Male | 57 | 71 | 178 | 22.41 |
| Participant 8 | Male | 58 | 64 | 165 | 23.51 |

**Table S2**. Top 18 significantly changed fecal microbiota with NMN supplementation in pre-aging mice.

| Phylum | Genus | Relative abundance, % | | | | | | |
| --- | --- | --- | --- | --- | --- | --- | --- | --- |
|  |  | Control group | | | NMN group | | | P value |
| Campilobacterota | Helicobacter | 4.206 | ± | 1.481 | 19.077 | ± | 4.778 | 0.040 |
| Proteobacteria | Psychrobacter | 3.335 | ± | 3.261 | 0.090 | ± | 0.045 | 0.033 |
| Desulfobacterota | Desulfovibrio | 2.203 | ± | 0.981 | 4.697 | ± | 2.839 | 0.031 |
| Firmicutes | Turicibacter | 1.584 | ± | 0.550 | 2.699 | ± | 1.414 | 0.007 |
| Verrucomicrobiota | Akkermansia | 2.223 | ± | 1.392 | 0.003 | ± | 0.003 | 0.000 |
| Deferribacteres | Mucispirillum | 0.031 | ± | 0.012 | 0.508 | ± | 0.256 | 0.009 |
| Firmicutes | Colidextribacter | 0.572 | ± | 0.093 | 0.802 | ± | 0.239 | 0.026 |
| unidentified_Bacteria | Candidatus_Saccharimonas | 0.355 | ± | 0.075 | 0.689 | ± | 0.180 | 0.036 |
| Firmicutes | Marvinbryantia | 0.042 | ± | 0.013 | 0.246 | ± | 0.184 | 0.052 |
| Firmicutes | Faecalibacterium | 0.000 | ± | 0.000 | 0.165 | ± | 0.145 | 0.033 |
| Firmicutes | Staphylococcus | 0.177 | ± | 0.141 | 0.033 | ± | 0.009 | 0.046 |
| unidentified_Bacteria | unidentified_Oscillospiraceae | 0.127 | ± | 0.029 | 0.363 | ± | 0.139 | 0.011 |
| Firmicutes | A2 | 0.004 | ± | 0.002 | 0.200 | ± | 0.111 | 0.023 |
| Actinobacteriota | Corynebacterium | 0.128 | ± | 0.117 | 0.025 | ± | 0.010 | 0.048 |
| Firmicutes | UCG-009 | 0.057 | ± | 0.010 | 0.246 | ± | 0.105 | 0.029 |
| Firmicutes | Oscillibacter | 0.093 | ± | 0.025 | 0.364 | ± | 0.116 | 0.005 |
| Proteobacteria | Paenalcaligenes | 0.104 | ± | 0.097 | 0.001 | ± | 0.001 | 0.034 |
| Firmicutes | Lachnospiraceae_UCG-001 | 0.009 | ± | 0.003 | 0.190 | ± | 0.107 | 0.000 |

The data present mean ± SEM. The significant difference between control and NMN group was conducted by student’s t-test at p<0.05 level.


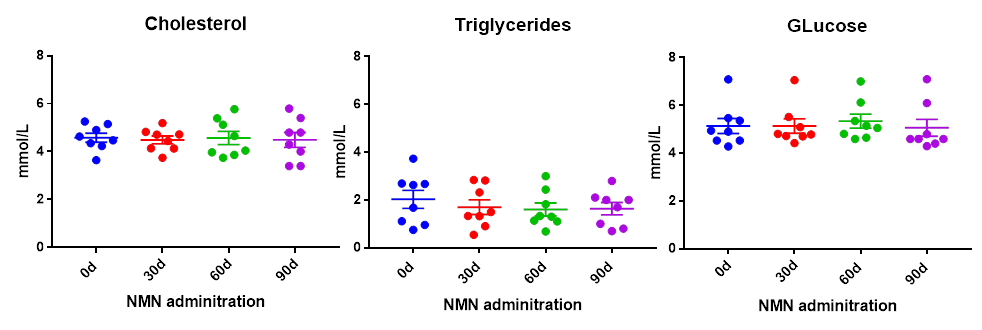


**Figure S1**. Serum cholesterol, triglycerides, and glucose contents in human volunteers after NMN administration at day 0, 30, 60 and 90.
